# Reply to Macbeth, F.; Treasure, T. Comment on “Pang et al. Ablative Techniques for Lung Metastases: Patient Selection and Outcomes Following Treatment with Stereotactic Radiotherapy or Radiofrequency Ablation. *Curr. Oncol.* 2025, *32*, 303”

**DOI:** 10.3390/curroncol32090518

**Published:** 2025-09-17

**Authors:** Nicos Fotiadis, Daniel Tong, Jennifer W. S. Pang, Merina Ahmed

**Affiliations:** 1 Royal Marsden NHS Foundation Trust, Downs Rd, Sutton SM2 5PT, UKjennifer.pang@nhs.net (J.W.S.P.);; 2 Maidstone and Tunbridge Wells NHS Trust, Maidstone Hospital, Hermitage Lane, Maidstone ME16 9QQ, UK

We thank Dr. Macbeth and Mr. Treasure for their interest in our article “Ablative Techniques for Lung Metastases: Patient Selection and Outcomes Following Treatment with Stereotactic Radiotherapy or Radiofrequency Ablation” published in *Current Oncology*. We welcome the opportunity to respond to their comments [1].

Our study was a retrospective analysis of a large single-institution cohort treated with either SABR or RFA for lung metastases of varied primary origin. As stated in our methods and discussion, the primary aim was to describe patterns of use, patient selection, and local treatment outcomes in a real-world setting, rather than to determine the definitive impact of these treatments on overall survival (OS) or quality of life (QoL).

We agree that high-quality randomized controlled trial (RCT) evidence is limited in this field, largely because the heterogeneity of primary tumours, metastatic patterns, and patient comorbidities poses challenges for trial design and recruitment. Nonetheless, the available data—including the SABR-COMET trial and its long-term follow-up—consistently suggest that in selected oligometastatic patients, local consolidative therapy improves progression-free survival (PFS) and may confer an OS benefit [2,3].

While the SABR-COMET trial had a modest sample size (n = 99), it demonstrated a sustained OS advantage at long-term follow-up (5-year OS 42.3% vs. 17.7%; HR 0.57) with acceptable toxicity [2,3]. Concerns about arm imbalance are acknowledged, but the survival difference persisted despite these factors. Similarly, the Gomez et al. trial in non-small-cell lung cancer, although stopped early due to the observed clinical efficacy, reported durable PFS and a clinically meaningful OS trend that aligns with other phase II signals [4].

A recently published meta-analysis of 15 RCTs and 1414 patients investigated the efficacy of metastasis-directed treatment (MDT) in oligometastatic disease [5]. This showed a statistically-significant improvement in both OS (HR 0.60, *p* < 0.001) and PFS (HR 0.48, *p* < 0.01) with MDT [5]. This provides further reassurance over the use of MDT while we await results from Phase III RCTs such as SABR-COMET10 (NCT03721341), SABR-COMET3 (NCT03862911), and OligoRARE (NCT04498767).

We respectfully disagree with the suggestion that PFS is an “unreliable outcome” in ablation trials. In oligometastatic disease, local progression often precipitates systemic progression and limits subsequent therapeutic options [6]. Durable local control can delay systemic therapy initiation, reduce symptom burden, and preserve QoL—outcomes that are clinically meaningful to patients even if OS benefit is not yet conclusively proven. Our own data demonstrate favourable local control rates with both SABR and RFA, consistent with other series [7,8].

Beyond the studies cited in our paper, multiple prospective series and meta-analyses have demonstrated that RFA achieves local control and survival outcomes comparable to SABR in selected patients, particularly for small peripheral lesions. For example, De Baère et al. reported a 4-year local efficacy of 89% for RFA of lung metastases < 4 cm [9], and Hasegawa et al. found a 3-year OS of 84% in a prospective multicentre study of colorectal lung metastases [10]. RFA remains particularly valuable when SABR is contraindicated, such as in patients with prior high-dose thoracic radiotherapy or centrally located lesions at high risk from re-irradiation.

We acknowledge the contribution of the PulMiCC trial [11], but it should be noted that it specifically addressed colorectal cancer lung metastases in a highly selected surgical population. Its applicability to our broader cohort—including sarcoma, renal, melanoma, and other primaries—is therefore limited. Furthermore, the trial was underpowered due to recruitment difficulties, and while the authors conclude that major survival benefit is “very unlikely,” the confidence intervals remain wide, leaving open the possibility of benefit in certain subgroups.

We respectfully disagree with the suggestion that SABR and RFA for asymptomatic oligometastatic lung disease should be paused outside of trials. In the absence of definitive RCT evidence, treatment decisions should be made through multidisciplinary discussion, weighing the available data, patient preferences, comorbidities, and the potential for local control to delay systemic progression. Pausing access could deny patients a chance at durable control, particularly in histologies where systemic options are limited.

In conclusion, our study provides valuable real-world data on treatment patterns and outcomes for lung metastases across multiple tumour types. While we agree that further high-quality RCTs are essential, the existing body of evidence—including our own—supports the continued judicious use of SABR and RFA in carefully selected patients.
